# Optimizing Tannin-NaCMC Compositions via DOE for Enhanced Carbon Yield and Strength in 3D-Printed Porous Carbon

**DOI:** 10.3390/polym17131859

**Published:** 2025-07-03

**Authors:** Wonseok Tae, Hao Cheng, Sangyou Kim, Yeongjun Lee, Wonsuk Jung

**Affiliations:** School of Mechanical Engineering, Chungnam National University, Daejeon 34134, Republic of Korea; baeksan400@o.cnu.ac.kr (W.T.); chenghao8@naver.com (H.C.); rlatkddb5@o.cnu.ac.kr (S.K.); kuronuma15844@gmail.com (Y.L.)

**Keywords:** 3D-printed porous carbon, tannin, sodium carboxymethyl cellulose, design of experiments, carbon yield, mechanical strength, additive manufacturing

## Abstract

We report the fabrication of lightweight porous carbon structures via UV-assisted photopolymerization molding using a commercial photocurable resin modified with natural tannin and sodium carboxymethyl cellulose (NaCMC) as sustainable additives. A systematic analysis was conducted by applying a Design of Experiments (DOE) approach and regression modeling to evaluate the effects of varying blend compositions on carbon yield and mechanical strength. The results indicate that increasing the tannin content led to a maximum carbon yield of 13.43%, with an average porosity of approximately 80% and a compressive strength around 1 kPa. NaCMC was found to effectively control the resin viscosity within printable limits of 0.2537 Pa·s, although NaCMC indirectly improved carbonization efficiency through normalized yield analysis. This work highlights the synergistic role of bio-based polymers in tuning porous carbon properties. The findings provide a data-driven framework for designing sustainable polymer-derived carbon materials, bridging additive manufacturing with green chemistry.

## 1. Introduction

Three-dimensional (3D) printing technology has emerged as an effective additive manufacturing method for fabricating intricate structures with complex geometries [1]. In recent years, its application has expanded across a wide range of research fields and industrial sectors [2,3]. Among various 3D-printing techniques, stereolithography (SLA) and digital light processing (DLP) stand out for their use of liquid photocurable resins, which are selectively polymerized upon exposure to ultraviolet (UV) or visible light, thereby enabling manufacturing processes with exceptionally high resolution and precision [4,5]. Compared to conventional subtractive manufacturing methods, these techniques offer significantly greater design freedom and flexibility. As a result, they have been effectively employed in the fabrication of functional materials with complex internal architectures using a variety of base materials, including biomaterials [6], polymers [7,8,9,10], ceramics [11], and metals [12], thereby broadening the scope of their applicability [13].

Carbon-based materials have found widespread applications across a range of fields, including electrochemical systems, catalyst supports, adsorbents, and biomedical devices [14,15,16]. Among these, porous carbon materials are of particular interest due to their high specific surface area and internal porosity, which significantly enhance their functional versatility. However, traditional fabrication methods for porous carbon structures often face considerable challenges, such as limited control over structural precision, complex processing routes, and the frequent reliance on precursors that impose substantial environmental burdens [17].

To address these limitations, the integration of bio-based carbon precursors with stereolithography (SLA) technology has been proposed as a promising strategy. In particular, tannin, a naturally occurring phenolic biopolymer, has been recognized as an attractive candidate due to its high carbon yield and excellent structural stability upon carbonization [18].

In the domain of 3D printing with natural bio-polymers, most prior works have focused on lignin, cellulose, or chitosan, typically within Fused Deposition Modeling (FDM) or Direct Ink Writing (DIW) systems, with limited application to SLA/DLP platforms [19]. Blyweert et al. demonstrated the feasibility of manufacturing bio-derived porous carbon architectures by printing tannin-based photocurable resins using SLA technology, followed by subsequent pyrolysis [17]. Furthermore, in their subsequent studies, they explored the optimization of post-treatment thermal processes to improve the oxidation resistance and mechanical properties of the printed carbon structures [20]. Notably, Blyweert et al. employed a Design of Experiments (DOE) approach to systematically investigate the effects of resin composition variations on the dimensional stability and carbon yield of the resulting structures [21]. Additionally, their work, involving the numerical modeling of pyrolysis reactions, highlighted the potential to predict structural evolution under different carbonization conditions [22].

While previous studies primarily focused on the formulation of photocurable resins or the proportion of a single carbon precursor, namely tannin, relatively few investigations have explored the use of commercially available resins or alternative carbon precursors. In particular, the incorporation of a large amount of tannin into hydrophobic resins often leads to the formation of finely dispersed phases, which significantly increases the viscosity of the resin mixture. This, in turn, can cause deterioration in printing quality and flowability during SLA processes [23,24]. To ensure the effective operation of SLA or DLP printing technologies and to achieve high-resolution structural fabrication, it is imperative to control the viscosity of the resin mixture within an appropriate range. Consequently, there is a clear need to consider alternative carbon precursor materials that can mitigate viscosity-related challenges while maintaining the printability and structural fidelity of the final constructs.

Therefore, in this study, a commercial photocurable resin was employed as the base matrix, and the amount of tannin was deliberately reduced to lower the viscosity of the resin mixture. To compensate for the reduced tannin content, sodium carboxymethyl cellulose (NaCMC) was introduced as an alternative additive. NaCMC is an anionic, water-soluble polymer derived from natural cellulose, and, owing to its high hydrophilicity, it readily dissolves in water and water-miscible alcohols, where it functions effectively as a thickener or stabilizer [25]. However, in hydrophobic organic solvents such as acrylate-based resins, NaCMC remains insoluble and exists as solid particles dispersed within the liquid matrix [26], thereby exerting a limited influence on the overall viscosity. Although NaCMC has been previously applied in various fields, such as the mechanical reinforcement of UF-based composites (İstek et al., 2020) and as a methacrylated hydrogel precursor (Giuseppe et al., 2020), due to its bio-based origin and functional versatility, its direct use as a carbon precursor in a photocurable resin system has not been reported [27,28]. Additionally, during pyrolysis, the reactive nature of the sodium element can be exploited to potentially enhance both the carbon yield and the compressive strength of the resulting carbon structures [29].

In this context, a DOE methodology was employed to systematically determine the optimal mixing ratios of tannin and NaCMC and to quantitatively analyze how their proportions affect the carbon yield and compressive strength of the final structures. Given that pyrolysis’ behavior—such as thermal decomposition and gas evolution patterns—directly impacts the shrinkage and integrity of the structures [30], a thermogravimetric analysis (TGA) was conducted for each component to establish appropriate pyrolysis temperature profiles. Post-carbonization, the porous carbon structures were quantitatively characterized in terms of porosity, carbon yield, and compressive strength, utilizing complementary techniques, including X-ray computed tomography (X-ray CT), helium pycnometry, and mechanical compression testing. Therefore, this study aims to develop a sustainable and printable precursor formulation for 3D-printed porous carbon by optimizing tannin and NaCMC compositions and to establish a data-driven framework that correlates precursor design with carbon yield and mechanical performance.

## 2. Materials and Methods

### 2.1. Materials

To fabricate the porous carbon structures, a base resin and two carbon-providing additives, tannin (tannic acid, C_76_H_52_O_46_, molecular weight 1701.20) and sodium carboxymethyl cellulose (NaCMC, C_6_H_7_O_2_(OH)_2_CH_2_COONa, molecular weight 250,000), were employed. The resin used as the base matrix was a commercially available Clear Resin (V4) supplied by Formlabs, which undergoes photopolymerization upon exposure to ultraviolet (UV) light at a wavelength of 405 nm. This resin was utilized to create carbon precursor mixtures by blending it with powder-form additives. The tannin utilized as a carbon precursor was extracted from Chinese natural gall nuts and procured from Sigma-Aldrich (St. Louis, MA, USA). Likewise, the NaCMC used in this study was also obtained from Sigma-Aldrich.

### 2.2. Manufacturing Process of 3D-Printed Carbon Precursor

**Figure 1:**
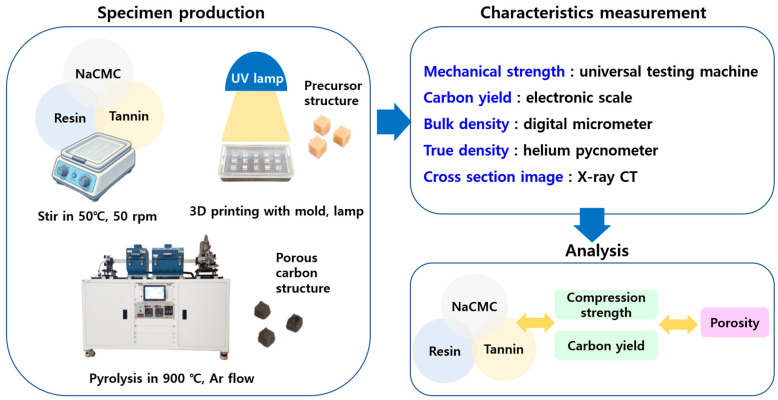
Experimental workflow for fabrication and characterization of tannin–NaCMC-based porous carbon structures.

The entire process, from specimen fabrication to characterization and analysis of the carbon structures, is presented in Figure 1.

The resin, tannin, and NaCMC were mixed according to the ratios determined by the DOE methodology. The resin was blended with tannin and NaCMC using a stirring hot plate (Jeio Tech, Daejeon, Korea) at 50 °C and a stirring speed of 50 rpm for 1 h. The resulting mixture was then poured into molds fabricated from polydimethylsiloxane (PDMS) for specimen preparation. Photopolymerization was performed by exposing the filled molds to a UV lamp (405 nm, 15 W) for 6 h. Subsequently, a post-curing step was carried out using a Form Cure device (Formlabs, Somerville, MA, USA) at room temperature for 1 h. The final specimens prepared for carbonization were cubic in shape, with each side measuring 6 mm in length. To minimize experimental variability, 35 specimens were fabricated for each experimental condition.

This mold-based approach was adopted to ensure consistent shape and volume for parametric analysis, serving as a preparatory step toward future implementation using DLP-based 3D printing with freeform geometries.

The carbonization process was carried out using a thermal chemical vapor deposition (CVD) system (Scen, Suwon, Gyeonggi Province, Korea) under an argon (Ar) atmosphere at a pressure of 10^−2^ Torr. The carbonization temperature profile was determined based on the thermogravimetric analysis (TGA) results of each material. According to the TGA measurements, the resin exhibited a significant weight loss around 352 °C, NaCMC showed a sharp decomposition at approximately 292 °C, and tannin exhibited notable thermal degradation at approximately 272 °C and 312 °C.

**Figure 2:**
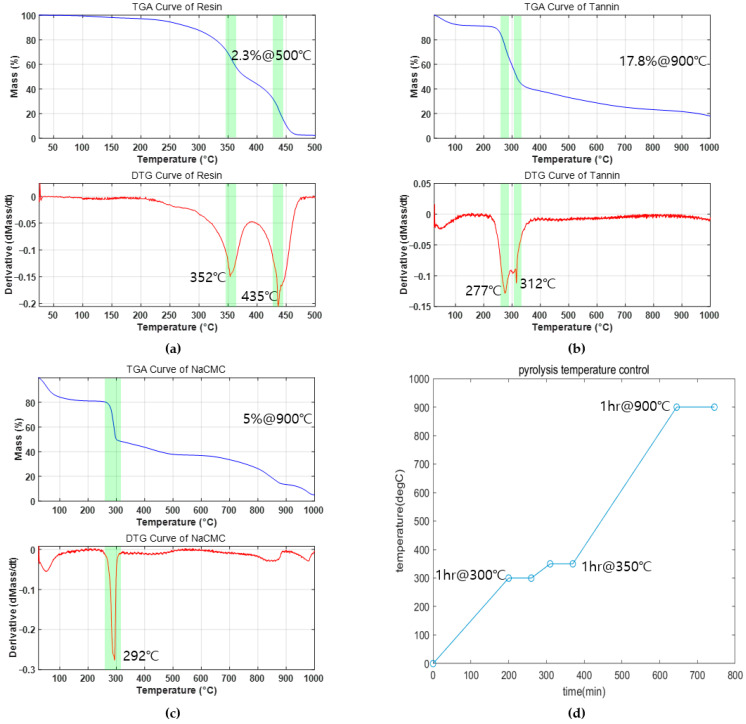
Thermogravimetric analysis of materials: (**a**) resin, (**b**) tannin, (**c**) and NaCMC, and (**d**) heating profile for pyrolysis.

To ensure the complete decomposition of all components, the carbonization temperature was programmed with three heating stages, each followed by an isothermal holding period. Initially, the temperature was ramped at a rate of 1.5 °C/min up to 300 °C and held at 300 °C for 1 h. Subsequently, the temperature was increased at 1 °C/min to reach 350 °C, where it was maintained for another 1 h. Finally, the temperature was ramped at 2 °C/min to a peak temperature of 900 °C, which was sustained for 1 h before natural cooling. The TGA results and the detailed carbonization temperature profile are presented in Figure 2.

### 2.3. Experimental Design

In this study, a DOE approach was employed to fabricate porous carbon structures based on a commercial photocurable resin and to systematically investigate their properties. DOE serves as an efficient methodology for analyzing interactions among multiple variables within complex systems, and it has been widely adopted in previous studies involving various mixture experiments. For quantitative analysis, Design-Expert 13 software (StatEase, Minneapolis, MN, USA) was utilized, and a simple lattice design for mixture experiments was selected to plan the experimental conditions.

Two key indicators were chosen to characterize the properties of the carbon structures: carbon yield and mechanical strength. The carbon yield was defined as the mass fraction of residual organic material remaining after high-temperature pyrolysis at 900 °C, representing the extent to which carbonaceous material was retained from the precursor. Carbon yield is a critical parameter that reflects the fundamental physical properties of the carbon precursor. Mechanical strength, a crucial property for practical applications of porous carbon structures, was assessed by measuring the compressive strength of the carbonized specimens. Compressive strength was selected because the intended applications—such as catalyst supports and electrode frames—require stability under compression rather than tensile loading, and because the high porosity and inherent microdefects of carbonized structures often impair the reliability of tensile or fracture-based tests.

To enable the high-resolution 3D printing of carbon precursors, it was essential to maintain the viscosity of the resin mixtures by incorporating tannin and NaCMC below a certain threshold. Accordingly, a constraint was imposed such that the combined content of tannin and NaCMC did not exceed 25 wt.%. Based on this criterion, a total of 13 experimental runs were planned.

Viscosity measurements revealed that the highest viscosity among the formulated mixtures occurred with a composition of 15% tannin and 10% NaCMC, yielding a viscosity of 0.2537 Pa∙s at a shear rate of 4000 s^−1^. This value is significantly lower than the 3 Pa∙s limit reported by Blyweert et al. for successful single-material stereolithographic printing [20], indicating that the resin mixtures used in this study were well within the acceptable range for photocuring-based 3D printing.

Natural biopolymers such as lignin, cellulose, and chitosan have predominantly been utilized in FDM or DIW platforms [19], where high viscosity and shear-thinning behavior are essential to ensure smooth extrusion and shape retention. In contrast, photocuring-based systems like SLA/DLP favor low and stable viscosity without shear-thinning, and the present formulation satisfies these requirements without additional chemical modification or rheological tuning.

The experimental design points are summarized in Table 1. A total of ten distinct formulation points were selected, comprising one center point, three vertex points, three center edge points, and three axial cubic points. In particular, considering the extreme mixture ratios associated with the vertex points, duplicate experiments were performed at each vertex condition. As a result, a total of 13 experimental runs were conducted.

## 3. Results and Discussion

### 3.1. Porosity Analysis of the Carbon Structure

To determine the bulk density, the average mass of 30 specimens prepared under each experimental condition was measured, along with the volume of each individual sample. The mean bulk density for each experimental point was then calculated by dividing the average mass by the average volume of the specimens. The true density was measured using an Accupyc II (Micromeritics, Norcross, GA, USA) helium pycnometer, with six measurements performed for each set of 30 samples to obtain a reliable average value. Based on the measured bulk and true densities, the porosity (φ) of each sample was calculated using Equation (1).

(1) $$\varphi =1-\frac{\rho_{bulk}}{\rho_{true}}$$

X-ray computed tomography (X-ray CT) measurements were conducted using a Skyscan 1272 system (Bruker AXS). The scans were performed at an acceleration voltage of 40 kV without a filter, with a rotation step of 0.5° and frame-averaging over 2 frames. High-resolution two-dimensional (2D) images with a resolution of 2048 × 2048 pixels were acquired, and the 2D datasets were subsequently reconstructed into three-dimensional (3D) images using Skyscan Ctvox software 3.3.1.

Mechanical testing was performed using a UNITEST M1 universal testing machine (Test One) to evaluate the compressive strength of the carbon structures. Compression tests were conducted at a loading rate of 2 mm/min, with the applied force and corresponding displacement recorded in real-time. The data were subsequently converted into compressive stress and strain values for analysis.

Electrical conductivity analysis was performed using a CMT-100 four-point probe system (AiT) to measure the sheet resistance $\left(R_{s}\right)$ of the carbonized specimens. The cubic samples (side length ≈ 5 mm) differ from the ideal thin-film geometry typically assumed in four-point probe models, requiring the application of shape- and edge-related correction factors. Bulk resistivity ρ was calculated using the following equation:

(2) $$\rho =R_{s}\cdot t\cdot \left(\frac{G_{Yilmaz}}{G_{ideal}}\right)\cdot F_{smits}$$

(3) $$\sigma =\frac{1}{\rho }$$

Here, t is the sample thickness, $G_{Yilmaz}$ is the geometric correction factor for finite sample thickness, $G_{ideal}$ is the ideal correction value for an infinite half-space geometry, and $F_{smits}$ accounts for the edge effects due to the sample-to-probe size ratio. Electrical conductivity σ was obtained as the reciprocal of ρ. Detailed parameter values and correction procedures are provided in the Supporting Information.

### 3.2. Properties of the Porous Carbon Structure

The average properties of the porous carbon structures fabricated with different compositions are summarized in Table 2. The carbon yield ranged from below 10% to a maximum value of 13.43%, with an average yield of approximately 10.8%. Specimens with relatively lower total amounts of tannin and NaCMC exhibited carbon yields below 10%. Compared to additional specimens fabricated solely with tannin or NaCMC, the blended formulations generally exhibited higher carbon yields. However, the compressive strength values were lower than that of the tannin-only specimen, but higher than the NaCMC-only specimen. Detailed results of these single-precursor compositions are provided in Table S1. The compressive strength averaged 0.945 kPa; however, compared to carbon yield, the compressive strength exhibited larger variations depending on the composition of the mixture.

**Figure 3:**
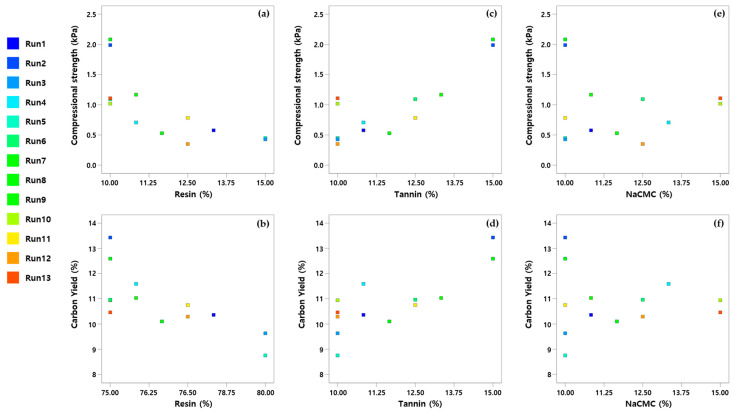
Correlation of material and carbon characteristics: (**a**) resin vs. compressional strength; (**b**) resin vs. carbon yield; (**c**) tannin vs. compressional strength; (**d**) tannin vs. carbon yield; (**e**) NaCMC vs. compressional strength; (**f**) NaCMC vs. carbon yield.

To investigate the linear correlations among variables, the Pearson correlation coefficients were calculated using the ‘correl’ function in Microsoft Excel. The correlation analysis between the mixture composition and the measured properties revealed that the amount of resin showed negative correlations with both carbon yield (correlation coefficient: −0.71) and compressive strength (correlation coefficient: −0.74). In contrast, the amount of tannin exhibited positive correlations with carbon yield (0.80) and compressive strength (0.83). Meanwhile, NaCMC content alone displayed weak negative correlations with carbon yield (−0.05) and compressive strength (−0.12). However, the combined amount of tannin and NaCMC showed positive correlations with carbon yield (0.74) and compressive strength (0.71). The correlation can be observed in Figure 3.

Table 3 presents the results of two different normalization approaches applied to the carbon yield to quantitatively evaluate the carbonization efficiency. The fifth column of Table 3 shows the carbon yield normalized by the weighted contributions of tannin and NaCMC, considering their respective residual ratios after high-temperature pyrolysis—17.8% for tannin and 5% for NaCMC, corresponding to a weight ratio of 1:0.28 (refer to Figure 2). This normalized value is presented under the column labeled “Carbon yield/Weighted Tannin.” The sixth column of Table 3 displays the carbon yield normalized solely by the mass fraction of tannin, excluding the contribution of NaCMC, and is denoted as “Carbon yield/Tannin.” These normalization methods adjust for the intrinsic differences in the carbon contributions of tannin and NaCMC depending on the mixture composition, allowing for an indirect assessment of the influence of NaCMC on the carbonization efficiency of tannin.

Examining the normalized values in the “Carbon yield/Tannin” column (sixth column) of Table 3 reveals that some experimental conditions (specifically runs 6, 7, and 9) exhibit values exceeding 1. This indicates that the achieved carbon yield surpasses what would be expected from the tannin content alone, thereby suggesting that the contribution of NaCMC to the final carbon yield cannot be disregarded.

Based on the normalization results, a comparison with the findings reported by Blyweert et al. was conducted. In the present study, the carbon yield normalized against the total carbon precursor content (fifth column) ranged from 0.66% to 0.85%, with an average value of 0.73%. In contrast, Blyweert et al. reported an average carbon yield of 18.84% [20]. When normalized against the tannin content (25 wt.%), their carbon yield values ranged from 0.65% to 0.86%, with an average of 0.75%. These results indicate that the carbonization efficiency observed in the present study is comparable to that reported in previous research. Although the direct correlation between NaCMC content and carbon yield appeared to be weak, the normalized yield analysis and compositional comparisons suggest that NaCMC plays a synergistic role in the carbonization process. Beyond its contribution as a carbon source through the formation of Na_2_CO_3_ residues, Na^+^ ions may act as catalysts that promote dehydration and condensation reactions during pyrolysis. These reactions can lead to enhanced cross-linking within the organic matrix, facilitating the retention of carbon. Moreover, the presence of Na^+^ can alter the thermal degradation pathway of tannin, potentially shifting it toward more char-forming mechanisms. These catalytic effects may become increasingly significant at higher NaCMC ratios or under modified thermal profiles, warranting further mechanistic investigations.

In previous studies, the carbon yield was optimized by varying the resin composition while keeping the tannin content constant, resulting in a normalized carbon yield range of 0.65–0.86%. In contrast, the present study employed a commercial resin, effectively eliminating variations associated with resin composition. Considering this distinction, it can be indirectly inferred that NaCMC contributed positively to the carbonization process and the resulting carbon yield.

During thermal decomposition, NaCMC undergoes decarboxylation of its –CH_2_COONa side groups, leading to the formation of Na_2_CO_3_. This inorganic residue remains as part of the solid fraction after pyrolysis, thus increasing the overall solid yield. Furthermore, Na^+^ ions act as alkaline catalysts during the pyrolysis of cellulose-based polymers, inhibiting the formation of volatile decomposition products while promoting the formation of fixed carbon (char), thereby contributing to a genuine enhancement in carbon yield [31,32]. Consequently, NaCMC simultaneously facilitates the retention of inorganic residues and the promotion of organic carbonization reactions, ultimately improving the overall carbonization efficiency.

**Figure 4:**
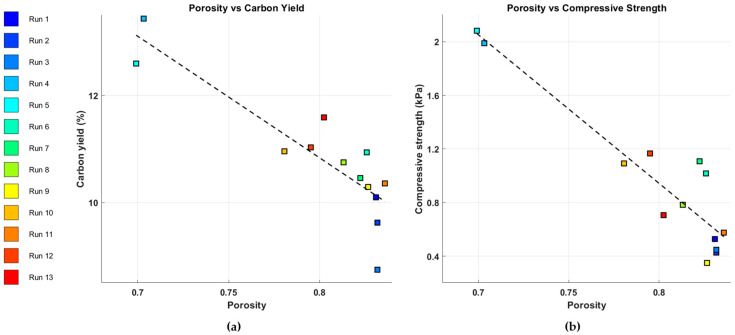
Correlation of porosity and (**a**) carbon yield or (**b**) compressional strength.

The bulk density varied depending on the mixture composition, while the true density ranged from 0.9220 g∙cm^−3^ to 1.2100 g∙cm^−3^. As shown in Figure 4, the correlation coefficients between the amount of resin and the bulk and true densities were −0.58 and −0.48, respectively, indicating negative correlations. In contrast, the correlation coefficients between the amount of tannin and the bulk and true densities were 0.88 and 0.48, respectively, demonstrating positive correlations. Meanwhile, the correlation coefficients between the NaCMC content and the densities were −0.30 for bulk density and 0 for true density, suggesting that NaCMC exhibited relatively weak or negligible correlations compared to resin and tannin.

Based on the measured bulk and true densities, the porosity (φ) of the samples was calculated and was found to range from 0.703 to 0.863. The correlation coefficient between porosity and carbon yield was −0.66, while that between porosity and compressive strength was −0.92, both indicating strong negative correlations.

A higher porosity indicates that a greater volume fraction within the structure is occupied by voids, which is attributed to the removal of the resin phase during the carbonization process. As the resin content increases, more resin is pyrolyzed, leading to an increase in the overall porosity of the final carbon structure. In porous materials, porosity acts as a major factor that directly influences compressive strength. Generally, structures with higher porosity exhibit a reduction in the effective load-bearing area per unit volume, which, in turn, leads to stress concentration around the pores. These localized stress concentrations serve as critical points for crack initiation and propagation, thereby weakening the mechanical integrity of the material [33].

To supplement this analysis, we performed a pore size distribution study using image-based segmentation. X-ray cross-sectional images of carbonized samples were binarized in MATLAB R2025a to isolate pore regions, and individual pore areas were measured and categorized into six size groups. Each size range was assigned a distinct color to visualize the spatial distribution of pores across the sample. As shown in Figure S1 and summarized in Table S2 (Supporting Information), more than 79% of the pores fell within the Size 0 and Size 1 categories (~50 μm). However, a non-negligible fraction of large pores (over 140 μm, Size 5) was also observed, indicating high structural heterogeneity despite similar bulk porosity. These morphological variations are likely to influence the mechanical response under compressive loading, supporting the interpretation that compressive strength is governed not only by porosity, but also by pore morphology.

However, in the high-porosity regime (φ > 0.80), the correlation between porosity and compressive strength becomes less pronounced. This deviation suggests that porosity alone does not fully capture the structural factors governing mechanical performance. At such high porosity levels, the mechanical integrity of the material is increasingly affected by pore morphology, including size, shape, spatial distribution, and interconnectivity. For example, larger or irregularly shaped pores can act as stress concentrators, while interconnected pore networks may reduce the effective load-bearing paths within the structure. These microstructural heterogeneities can lead to localized mechanical failures, even when the overall porosity remains constant. Therefore, compressive strength in highly porous carbon structures should be interpreted as a function of both the total porosity and morphological characteristics of the pore network.

The compressive strengths presented in Table 2 are significantly lower than those reported in previous studies. This discrepancy can be attributed to three main factors. The first is the difference in porosity. While the average porosity in previous studies was approximately 0.75, the structures fabricated in this study exhibited a higher average porosity of around 0.8. Under compressive loading, pores disrupt the uniform distribution of internal stresses and tend to accelerate structural collapse. It has been reported that compressive strength decreases exponentially with increasing porosity [33].

The second factor is the influence of sodium (Na) content. In general, carbonized structures derived from NaCMC-based precursors benefit from the presence of Na, which promotes the cross-linking of the carbon skeleton and facilitates the formation of a denser and more continuous amorphous carbon network during high-temperature carbonization [26]. However, excessive or unevenly distributed inorganic salts can introduce pore defects and microcracks, which negatively affect compressive strength. Therefore, the amount of NaCMC added and its uniform dispersion are critical factors [34]. In this study, the NaCMC content relative to the tannin content appears to have contributed negatively to the compressive strength of the carbon structures.

Finally, the compressive strength of porous structures is influenced not only by overall porosity, but also by the size, distribution, and connectivity of pores. Larger or unevenly distributed pores induce stress concentrations, making the structures more susceptible to failure. Moreover, higher pore connectivity reduces the number of effective load-bearing paths, leading to structural weakening. Consequently, differences in the internal architecture between the carbon structures fabricated in the two studies likely contributed to the observed differences in mechanical strength [35]. Generally, there is an inherent trade-off between porosity and compressive strength, requiring a careful balance depending on the target performance. However, it should be noted that, through the optimal control of pore size and distribution, it may be possible to partially improve both properties simultaneously.

**Figure 5:**
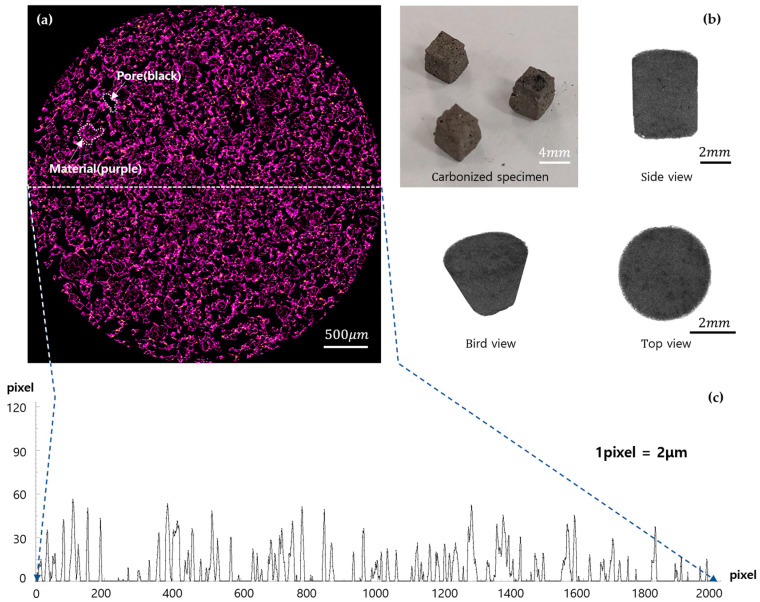
(**a**) Cross-sectional X-ray image; (**b**) 3D image synthesized using 2D X-ray images; (**c**) profile of the center of the cross-section (white line).

X-ray CT images of the specimens are presented in Figure 5. Figure 5a shows a cross-sectional image taken at a specific depth within the specimen. In the image, the regions colored red represent areas where carbon material remains, while the black regions correspond to voids, indicating the formation of pores. The pore sizes were observed to range from 20 to 350 μm, and they appeared to be uniformly distributed across the cross-section, as shown in Figure 5c. However, in some regions, localized variations in pore size were also identified, which may have originated from the heterogeneous dispersion of carbon precursors such as NaCMC or tannin during resin mixing. This non-uniformity could potentially contribute to local differences in polymerization and carbonization behavior. No significant differences were observed between samples, and similar cross-sectional morphologies and pore size distributions were identified across all specimens. Figure 5b presents a three-dimensional (3D) reconstructed image generated by stacking tomographic slices taken at different depths. The 3D visualization reveals that, after carbonization, the specimens exhibited a highly porous structure resembling a sponge.

On the other hand, the dispersion state of NaCMC within the hydrophobic resin matrix could significantly influence the final structure. Given that NaCMC is insoluble in the acrylate-based resin and exists as solid particulates, local agglomeration or non-uniform distribution may lead to micro-defects or regions of inconsistent cross-linking density. These heterogeneities may not only affect the mechanical integrity of the carbonized structures, but also introduce variations in porosity and shrinkage during pyrolysis. Although the average pore morphology appeared consistent across samples, as revealed using X-ray CT, localized effects from uneven NaCMC dispersion may contribute to variability in compressive strength, particularly in compositions with high NaCMC content.

Despite the relatively low compressive strength observed in the carbonized structures primarily attributed to their high porosity and localized defects induced during pyrolysis their ultralightweight and highly porous architecture (porosity > 80%) offers clear advantages for passive applications. Such structures are well-suited for use as electrochemical catalyst supports, VOC filters, and gas adsorption/separation systems, where mechanical loading is minimal [36]. Furthermore, the use of bio-based and biodegradable precursors, such as tannin and NaCMC, expands the material’s utility toward transient or sacrificial applications, particularly in biomedical fields. For instance, NaCMC’s widespread use in hydrogels and tissue scaffolds highlights the feasibility of employing these carbon structures in implantable or environmentally degradable platforms, especially where structural stability is only temporarily required.

The low compressive strength, significant structural shrinkage during carbonization, and absence of electrical property characterization currently preclude direct implementation in load-bearing or precision-critical environments. Thus, the present work should be regarded as a foundational study focused on material feasibility rather than immediate structural deployment.

Using the measured sheet resistance and applying the appropriate correction factors, the bulk resistivity and electrical conductivity were calculated for each formulation and are summarized in Table S3. The resulting conductivities ranged from 9.41 to 17.22 S/cm, exceeding the previously reported value of 7.1 S/cm by Blyweert et al., and demonstrating that the carbonized structures developed in this study exhibit sufficient conductivity for potential use in electrochemical catalyst supports or electrode components.

### 3.3. Results of the Experimental Design and Statistical Analysis

Using the DOE approach, regression models were developed to describe the relationships between the mixing ratios of resin, tannin, NaCMC, and the resulting carbon yield and compressive strength of the three-dimensional porous carbon structures. Two separate regression equations were generated, with the resin content, tannin content, and NaCMC content serving as independent variables. A Scheffé model, which is appropriate for mixture regression analysis, was employed to construct the equations. The resulting regression functions are presented in Equations (4) and (5).

(4) Compressive strength=0.4611 A+2.03 B+1.06 C −1.72 AB−1.49 AC−1.88 BC

(5) Carbon yield=9.23 A+12.55 B+10.73 C

The regression equation for compressive strength is expressed as a second-order polynomial. In the model, A represents the mass fraction of resin (wt.%), B represents the mass fraction of tannin (wt.%), and C represents the mass fraction of NaCMC (wt.%). The *p*-value for the model was found to be less than 0.0001, indicating a high level of statistical significance. The second-order terms representing the interactions between the components were also statistically significant. Furthermore, the lack-of-fit *p*-value was 0.1753, suggesting that the lack of fit was not statistically significant, and, thus, the second-order model exhibited a sufficiently high goodness of fit with the experimental data.

In contrast, the regression equation for carbon yield was expressed as a first-order polynomial. The model demonstrated strong statistical significance with a *p*-value of 0.0004 and was deemed appropriate for describing the carbon yield behavior. The lack of fit *p*-value was 0.4323, indicating that the model adequately fit the experimental data. More detailed statistical validation results for the regression models are summarized in Table 4.

**Figure 6:**
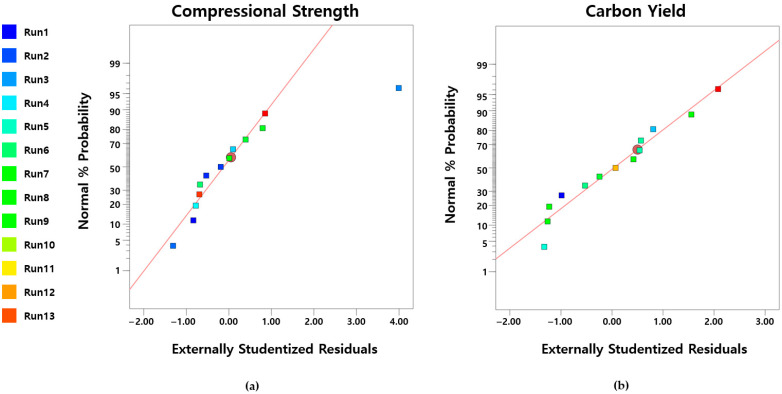
Normal plot of residuals: (**a**) compressional strength; (**b**) carbon yield.

The normal probability plots of the residuals for the compressive strength and carbon yield responses are shown in Figure 6. Although the first experimental data point for compressive strength appears to be an outlier, the majority of the experimental points for both carbon yield and compressive strength are closely aligned along the reference line. This indicates a reliable agreement between the experimental results and the predicted values from the regression models.

**Figure 7:**
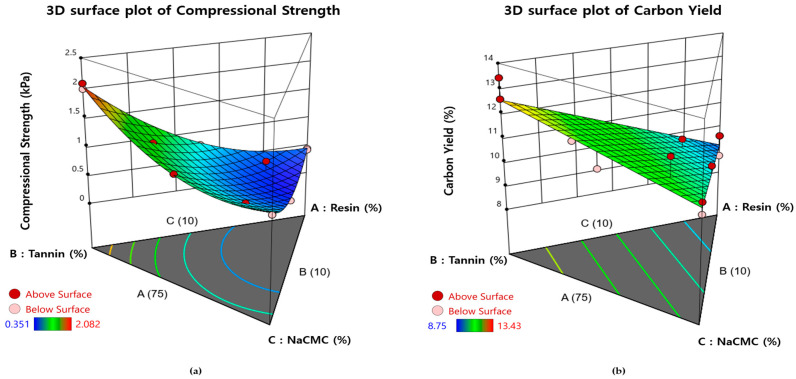
Contour plot (top) and surface plot (bottom) of the effect of composition ratio: (**a**) compressional strength; (**b**) carbon yield.

The relationships between the material composition ratios and the resulting compressive strength and carbon yield were further analyzed using three-dimensional contour plots. As shown in Figure 7, an increase in the resin content within the mixture leads to a decrease in both compressive strength and carbon yield. This trend indicates that, in order to fabricate porous carbon structures with sufficient mechanical strength, it is necessary to reduce the resin content while increasing the tannin content.

While the regression models demonstrated good goodness-of-fit within the designed experimental conditions, validation over a broader compositional range would be required to extend their predictive applicability. Furthermore, although the models independently describe compressive strength and carbon yield, these two properties are not truly independent in practice. For real-world applications, it is essential to simultaneously consider both mechanical and carbonization performance when optimizing the material formulation.

## 4. Conclusions

In this study, the effects of the mixing ratios of commercially available photocurable resin, tannin, and NaCMC on two response variables—compressive strength and carbon yield—were investigated using DOE and regression analyses. A total of 13 experiments were conducted, and systematic interaction analyses among the composition variables were performed based on the Scheffé model, leading to the development of first- and second-order regression equations. Both models were found to be statistically significant. Although the addition of NaCMC did not show a strong direct correlation with compressive strength or carbon yield, comparisons with normalized carbon yield values from previous studies suggested that NaCMC contributed positively to improving the carbonization efficiency.

This study demonstrates the potential for the effective fabrication of porous carbon structures with complex geometries through the integration of biomass-based precursors and 3D-printing technology. By proposing a carbonization process based on the adjustment of precursor composition, practical guidelines for the manufacturing process were established. These findings enhance the accessibility of porous carbon structure fabrication, which has traditionally been challenging, and highlight the potential for utilizing alternative components beyond tannin.

Several limitations of the present study should be acknowledged. First, the range of tannin and NaCMC mixture ratios was constrained by viscosity requirements, limiting the exploration of extreme conditions with high precursor contents. As a result, compositions involving pure tannin or pure NaCMC were not investigated. Second, the analysis of mechanical strength was primarily focused on porosity as the influencing factor. Although compressive strengths were significantly lower than those reported in previous studies, a more detailed investigation into morphological characteristics, such as pore size, distribution, and connectivity, is needed. Third, the optimal range of NaCMC content that could simultaneously enhance both carbonization efficiency and mechanical strength was not clearly determined.

To address the limitations identified in this study, future work will pursue multiple research directions aimed at improving the performance, structural integrity, and practical applicability of bio-based porous carbon structures. First, the compositional space will be expanded to increase the carbon precursor content while maintaining printability through optimized formulation strategies. The mixing ratio of NaCMC will be extended, and ultrasonic dispersion techniques will be introduced to improve particle distribution uniformity. The effects of NaCMC’s particle size, dispersion state, and local agglomeration on mechanical strength and pore architecture will be quantitatively assessed using high-resolution tools such as SEM and EDS.

Second, detailed X-ray CT and cross-sectional image analyses will be performed to develop predictive models that link internal pore morphology with compressive strength. In addition, a new DOE approach will be employed to investigate the influence of carbonization conditions, such as heating rate, isothermal dwell time, and peak temperature, on material properties. Multi-objective optimization strategies, such as Pareto front and the weighted-sum method, will be applied to identify the best balance between porosity and compressive strength.

Third, mechanical performance will be enhanced by incorporating heat-resistant or high-strength resins and nanoscale reinforcements such as carbon nanotubes. Dog-bone–shaped specimens will be fabricated to evaluate tensile strength and fracture toughness. Simultaneously, key DLP printing parameters, such as layer thickness, exposure time, and printing speed, will be optimized to ensure dimensional precision and structural reliability in complex-shaped specimens.

Finally, environmental durability and long-term stability will be assessed under various conditions, including temperature, humidity, and chemical exposure. The safety of volatile compounds and Na-based residues released during pyrolysis will be quantitatively evaluated using TGA-FTIR and ICP analyses. Collectively, these efforts will advance our understanding of the structural performance of porous carbon materials and support their industrial scalability, environmental safety, and functional versatility in emerging applications.

## Figures and Tables

**Table 1:** Experimental design of resin-based compound.

Exp. No.	Space Type	Resin (%)	Tannin (%)	NaCMC (%)
1	Center	76.6667	11.6667	11.6667
2	Vertex	80	10	10
3	Vertex	80	10	10
4	Vertex	75	15	10
5	Vertex	75	15	10
6	Vertex	75	10	15
7	Vertex	75	10	15
8	CenterEdge	77.5	12.5	10
9	CenterEdge	77.5	10	12.5
10	CenterEdge	75	12.5	12.5
11	AxialCB	78.3333	10.8333	10.8333
12	AxialCB	75.8333	13.3333	10.8333
13	AxialCB	75.8333	10.8333	13.3333

**Table 2:** Experimental results for the carbon specimen by mixture design.

Exp. No.	Carbon Yield (%)	Bulk Density (g/cm^3^)	True Density (g/cm^3^)	Porosity	Compressive Strength (kPa)
1	10.10	0.1568	0.9280	0.8310	0.529
2	9.63	0.1698	1.0100	0.8319	0.428
3	8.75	0.1683	1.0100	0.8319	0.449
4	13.43	0.3532	1.1900	0.7032	1.988
5	12.59	0.3521	1.1700	0.6991	2.082
6	10.94	0.2019	1.1600	0.8260	1.018
7	10.46	0.2023	1.1400	0.8225	1.108
8	10.75	0.1979	1.0600	0.8133	0.783
9	10.29	0.1598	0.9220	0.8267	0.351
10	10.96	0.2216	1.0100	0.7806	1.093
11	10.36	0.1886	1.1500	0.8360	0.577
12	11.03	0.2480	1.2100	0.7951	1.167
13	11.59	0.2054	1.0400	0.8025	0.707

**Table 3:** Carbon yields and normalized carbonization efficiencies of carbon precursor blends composed of tannin and NaCMC.

Exp. No.	Tannin (%)	NaCMC (%)	Carbon Yield (%)	Carbon Yield/Weighted Tannin	Carbon Yield/Tannin
1	11.6667	11.6667	10.10	0.68	0.87
2	10	10	9.63	0.75	0.96
3	10	10	8.75	0.68	0.88
4	15	10	13.43	0.70	0.90
5	15	10	12.59	0.66	0.84
6	10	15	10.94	0.85	1.09
7	10	15	10.46	0.82	1.05
8	12.5	10	10.75	0.67	0.86
9	10	12.5	10.29	0.80	1.03
10	12.5	12.5	10.96	0.68	0.88
11	10.8333	10.8333	10.36	0.75	0.96
12	13.3333	10.8333	11.03	0.65	0.83
13	10.8333	13.3333	11.59	0.84	1.07

**Table 4:** ANOVA results.

Source	Sum of Squares	Degree of Freedom	Mean Square	F-Value	*p*-Value
ANOVA Compressive Strength					
**Model**	3.69	5	0.7388	109.42	<0.0001
Linear mixture	3.03	2	1.52	224.75	<0.0001
AB	0.1860	1	0.1860	27.55	0.0012
AC	0.1408	1	0.1408	20.85	0.0026
BC	0.2234	1	0.2234	33.09	0.0007
**Residual**	0.0473	7	0.0068		
Lack of fit	0.0386	4	0.0096	3.33	0.1753
Pure error	0.0087	3	0.0029		
**Corrected Total**	3.74	12			
ANOVA Carbon Yield					
**Model**	13.86	2	6.93	19.24	0.0004
Linear mixture	13.86	2	6.93	19.24	0.0004
**Residual**	3.60	10	0.3602		
Lack of fit	2.75	7	0.3923	1.38	0.4323
Pure error	0.8552	3	0.2851		
**Corrected Total**	17.46	12			

## Data Availability

The data presented in this study are available on request from the corresponding author.

## Supplementary Materials

The following supporting information can be downloaded at: https://www.mdpi.com/article/10.3390/polym17131859/s1, Figure S1: (a) X-ray image of carbonized structure (b) Pore size distribution map.; Table S1:Experimental Validation of Tannin and NaCMC as Single Carbon Sources.; Table S2. Number of Pores Classified by Size Range; Table S3. Bulk resistivity and calculated electrical conductivity for each mixing composition, corrected using Smith and Yilmaz factors.; Table S4. Viscosity values for three resin–tannin–NaCMC formulations.
