# Supplementary material for: Optimizing Tannin-NaCMC Compositions via DOE for Enhanced Carbon Yield and Strength in 3D-Printed Porous Carbon
Source: Polymers (Basel). 2025 Jul 3;17(13):1859. doi: 10.3390/polym17131859 (PMC12251910; doi:10.3390/polym17131859)
Supplement: Supplementary file 1 [file polymers-17-01859-s001.zip › polymers-3708705-supplementary.pdf]

Supporting Information

1. Additional Experiments Using Single-Component Precursor Formulations

We conducted supplementary experiments using these extreme formulations for comparison with the original mixed compositions.

Table S1. Experimental Validation of Tannin and NaCMC as Single Carbon Sources

Exp. No.	Resin	Carbon precursor	Carbon yield (%)	Compressive strength (kPa)
14(Tannin only)	75 wt.%	25 wt.%	7.25	3.99
15(NaCMC only)	75 wt.%	25 wt.%	6.46	0.69

Experimental results indicated that, in terms of carbon yield, the use of pure tannin or pure NaCMC resulted in lower yields compared to the mixed formulations. With respect to compressive strength, the formulation containing only NaCMC exhibited intermediate strength levels within the range of the mixed compositions, whereas the formulation containing only tannin demonstrated even higher compressive strength than any of the mixed compositions.

2. Pore size distribution analysis using Matlab

We performed a pore size distribution analysis using Matlab. Binary segmentation was applied to X-ray cross-sectional images of the carbonized structures to extract pore regions. Based on the calculated area (in pixels) of each detected pore, we categorized the pores into six size ranges:

Each size range was assigned a distinct color to enable intuitive visual identification. Representative results are presented in the following figure and table.

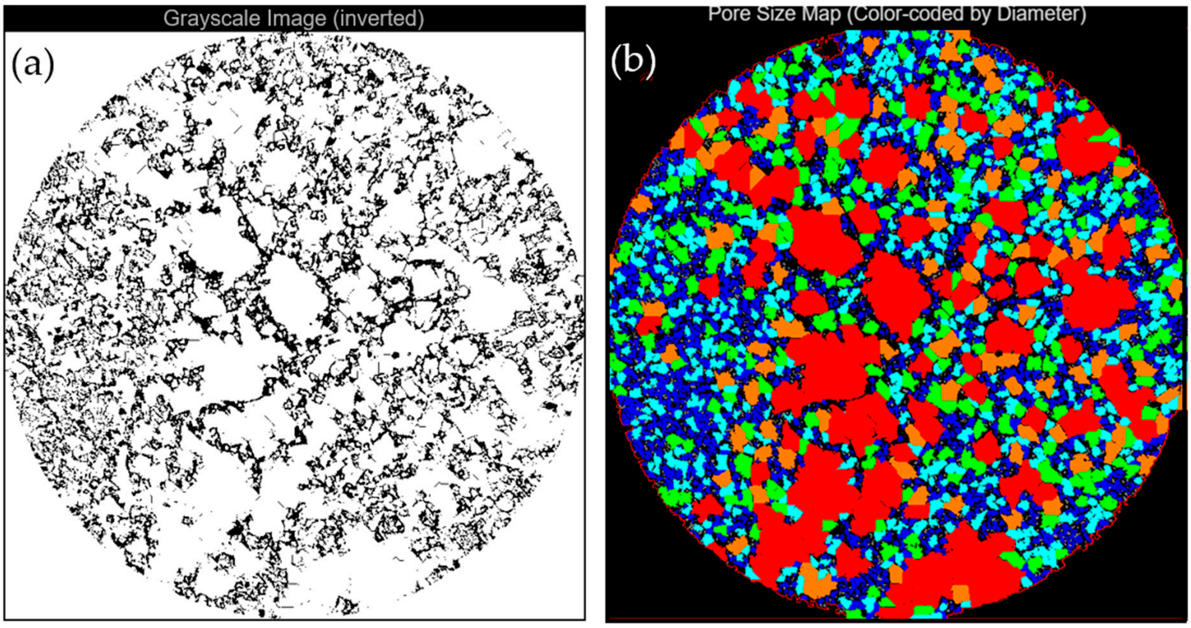

Figure S1. (a) X-ray image of carbonized structure (b) Pore size distribution map

As shown in Figure S1 and Table S2, most pores fell within the Size 0 and Size 1 category (~50 μm), occupying over 79% of the total. However, a non-negligible fraction of larger pores (Size 5) was also present, indicating high structural heterogeneity. Table S1 summarizes the quantitative results of pore counts and distributions for each size category.

Table S2. Number of Pores Classified by Size Range

Specimen	size0	size1	size2	size3	size4	size5	total
	~20μm	20~50μm	50~80μm	80~110μm	110~140μm	140~μm	
sample0	1388	2139	629	183	71	117	4527
sample1	1457	1851	437	164	84	150	4143
sample2	1946	3591	901	225	73	43	6779
sample3	1600	3277	941	246	87	51	6202
sample4	1589	3278	950	245	87	51	6200
sample5	1708	2021	475	188	73	121	4586
sample6	1687	3708	1099	230	63	34	6821
sample7	1687	3708	1099	230	63	34	6821
sample8	1297	2060	480	182	94	126	4239
sample9	1722	2077	570	217	99	118	4803
sum	16081	27710	7581	2110	794	845	55121
ratio	29.17%	50.27%	13.75%	3.83%	1.44%	1.53%	100.00%

3. Electrical Conductivity and Bulk Resistivity Analysis

Although the main focus of this study was the optimization of tannin–NaCMC composition in relation to carbon yield and compressive strength, the potential applications of porous carbon structures in electrochemical catalysis or electrodes require evaluation of their electrical characteristics.

Accordingly, we measured the sheet resistance of carbonized samples using a CMT-100 (AiT) four-point probe system, and converted these values to bulk resistivity and electrical conductivity by applying correction factors for sample geometry:

The cubic bulk samples (side ~5 mm) differ from the ideal assumptions used in flat sheet models. Therefore, we introduced shape correction factors based on established models:

$$F_1(t/s) = \frac{t/s}{2\ln\left(\frac{\sinh(t/s)}{\sinh(t/2s)}\right)} \approx 0.999$$

F_smith = 1.7 (for edge effect, based on sample-to-probe size ratio)

$$G_{Yilmaz} = 2\pi \cdot s \cdot F_1(t/s) = 2\pi \cdot 0.05 \cdot 0.999 \approx 0.314$$

G_yilmaz = 0.314 (for finite thickness, calculated from 5 mm sample thickness and 0.5 mm probe spacing)

G_ideal = 4.532 (ideal correction factor for infinite medium)

$$\rho = R_s \cdot t \cdot \frac{G_{Yilmaz}}{G_{ideal}} \cdot F_{smith}$$

$$\sigma = \frac{1}{\rho}$$

Using these, we calculated the bulk resistivity and electrical conductivity for each formulation, summarized in Table S3 (see attached). The measured conductivities ranged from 9.41 to 17.22 S/cm, which notably exceed the value reported by Blyweert et al. (7.1 S/cm), and indicate that the current system is sufficiently conductive for use in electrochemical catalyst supports or electrode components.

Table S3. Bulk resistivity and calculated electrical conductivity for each mixing composition, corrected using Smith and Yilmaz factors.

Composition	Sheet resistance (Ω/sq)	Thickness (mm)	Bulk resistivity ($\Omega \cdot$ cm)	Conductivity (S/cm)
0	1.257	0.513	0.076	13.18
1	1.390	0.505	0.083	12.09
2	1.554	0.498	0.091	10.98
3	1.439	0.510	0.086	11.57
4	1.477	0.508	0.088	11.33
5	1.770	0.510	0.106	9.41
6	1.027	0.480	0.058	17.22
7	1.511	0.500	0.089	11.24
8	1.166	0.500	0.069	14.57
9	1.236	0.520	0.076	13.21

[S1] Smits, F.M. Measurement of Sheet Resistivities with the Four-Point Probe. Bell Syst. Tech. J. 1958, 37, 711–718. https://doi.org/10.1002/j.1538-7305.1958.tb03883.x

[S2] Yilmaz, S. The Geometric Resistivity Correction Factor for Several Geometrical Samples. J. Semicond. 2015, 36, 082001. https://doi.org/10.1088/1674-4926/36/8/082001.

4. Probing Viscosity of Representative Resin Formulations

Three compositions, each containing 75 wt.% commercial resin and varying ratios of tannin and NaCMC, exhibited viscosities in the range of 0.2483–0.2537 Pa·s when measured at a shear rate of 4000 s^{-1}. These values are significantly below the 3 Pa·s thresholds commonly accepted for continuous printing in vat photopolymerization systems, confirming their suitability for DLP/SLA printing platforms.

Table S4. Viscosity values for three resin–tannin–NaCMC formulations.

Composition	Resin	Tannin	NaCMC	viscosity
1	75 wt.%	12.5 wt.%	12.5 wt.%	0.2527Pa•s
2	75 wt.%	15 wt.%	10 wt.%	0.2537Pa•s
3	75 wt.%	10 wt.%	15 wt.%	0.2483Pa•s
